# Supplementary material for: Gut Microbiota Profiling as a Promising Tool to Detect Equine Inflammatory Bowel Disease (IBD)
Source: Animals (Basel). 2024 Aug 18;14(16):2396. doi: 10.3390/ani14162396 (PMC11350833; doi:10.3390/ani14162396)
Supplement: Supplementary file 1 [file animals-14-02396-s001.zip › Sävilammi et al. SupplementaryFigures.pdf]

Supplementary figures

Gut Microbiota Profiling as a Promising Tool to Detect Equine Inflammatory Bowel Disease (IBD)

Tiina Sävilammi, Rinna-Riikka Alakangas, Tuomas Häyrynen and Silva Uusi-Heikkilä

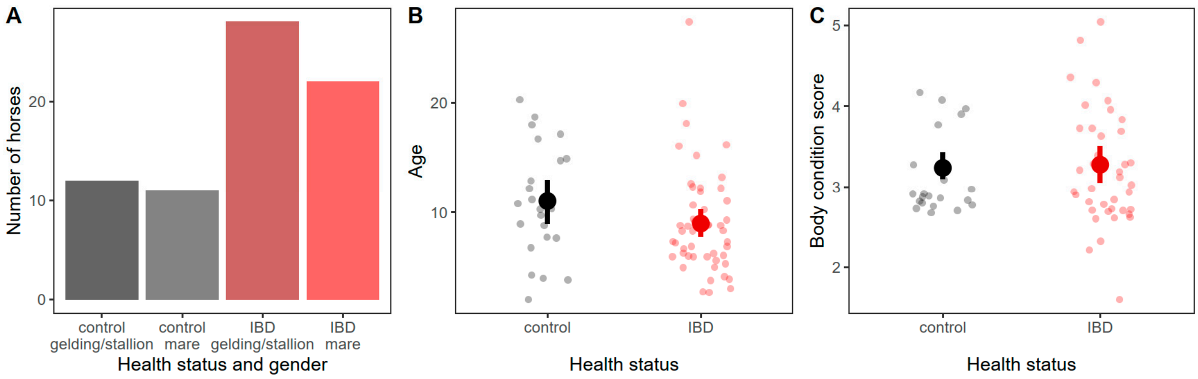

Figure S1. Background information of gender, age and body condition of horses used in the study. (A) Number of horses per gender, (B) age of horses and (C) body condition score of horses in both health status group (i.e., control and IBD; survey IBD- and acute IBD-horses combined). Group-wise means indicated with filled symbols and non-parametric 95% confidence intervals with whiskers.

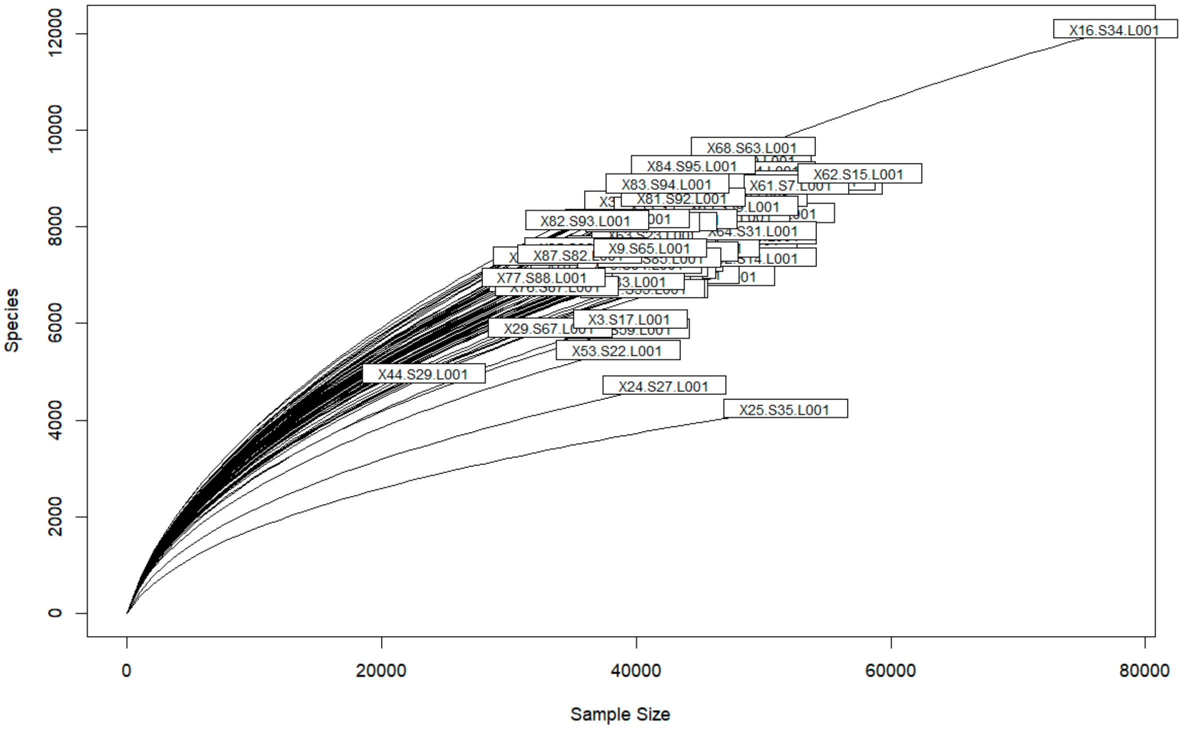

Figure S2. Rarefication curves of 76 samples of paired-end reads, sequenced aiming up to 100,000 pairs.

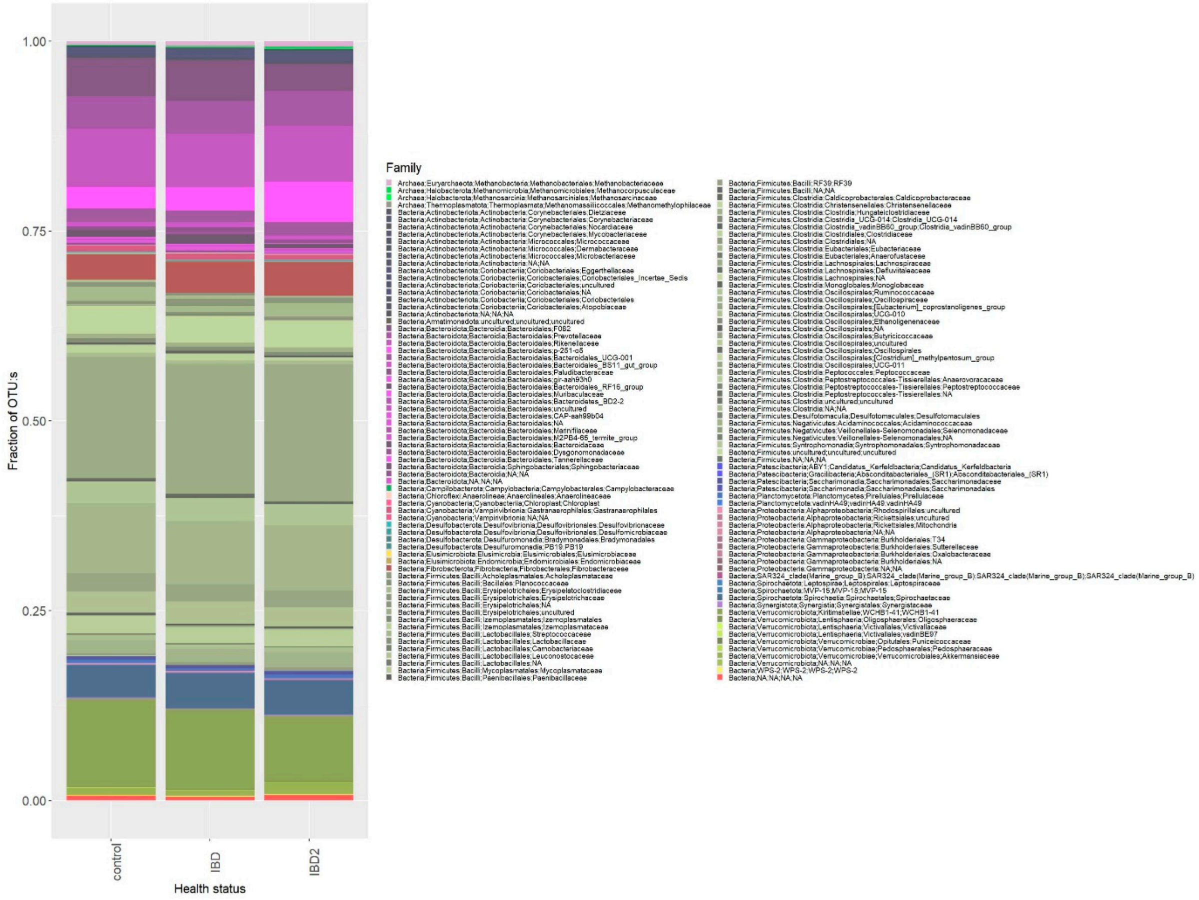

Figure S3. Family-level abundancies of all observed OTUs in control, survey-IBD and acute-IBD groups of horses. Rarefied counts were summed together among all individuals in each group.

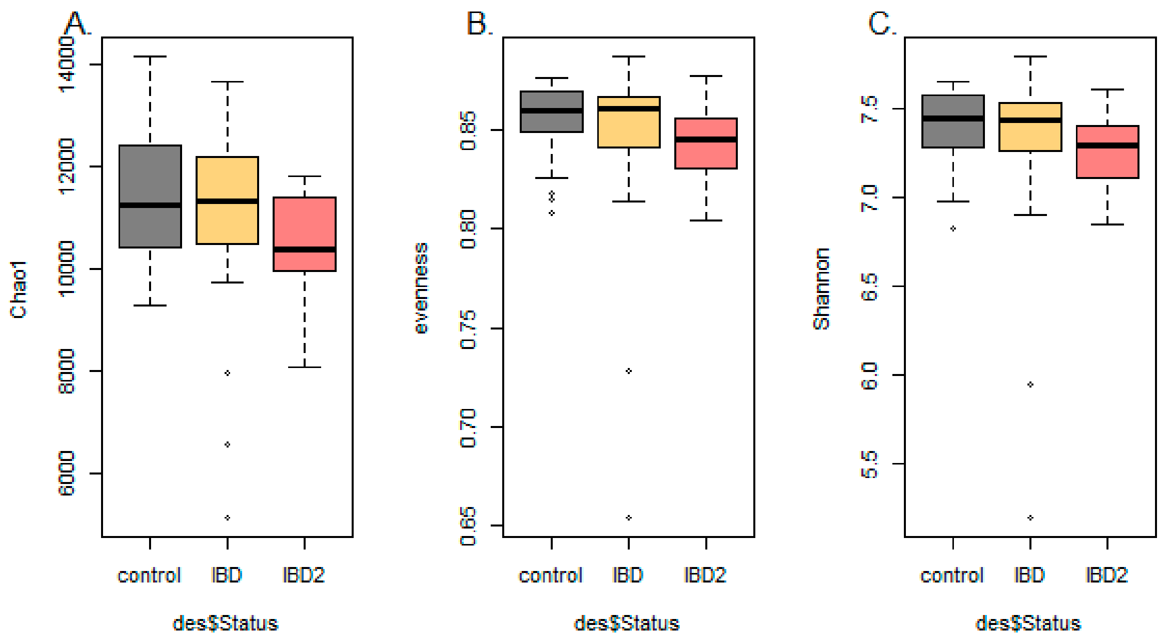

Figure S4. Chao1 (A), evenness (B) and Shannon (C) estimates of the within-individual diversity indices of control (black), survey-IBD (orange) and acute-IBD (red) health status groups of horses. The averages of alpha diversities were measured from rarefied data and did not deviate between any of the groups in any of the comparisons using ANOVA with $P<0.05$.

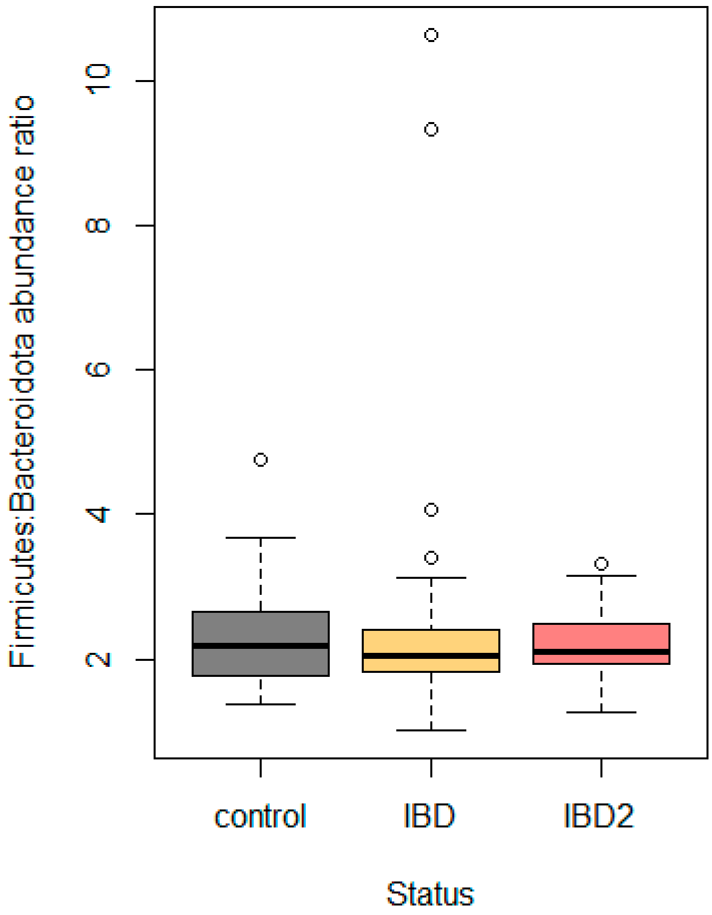

Figure S5. Total Firmicutes:Bacteroidetes abundance ratio (rarefied reads mapped to Firmicutes species : rarefied reads mapped to Bacteroidota species) among Control (black), survey-IBD (orange) and acute-IBD horses.

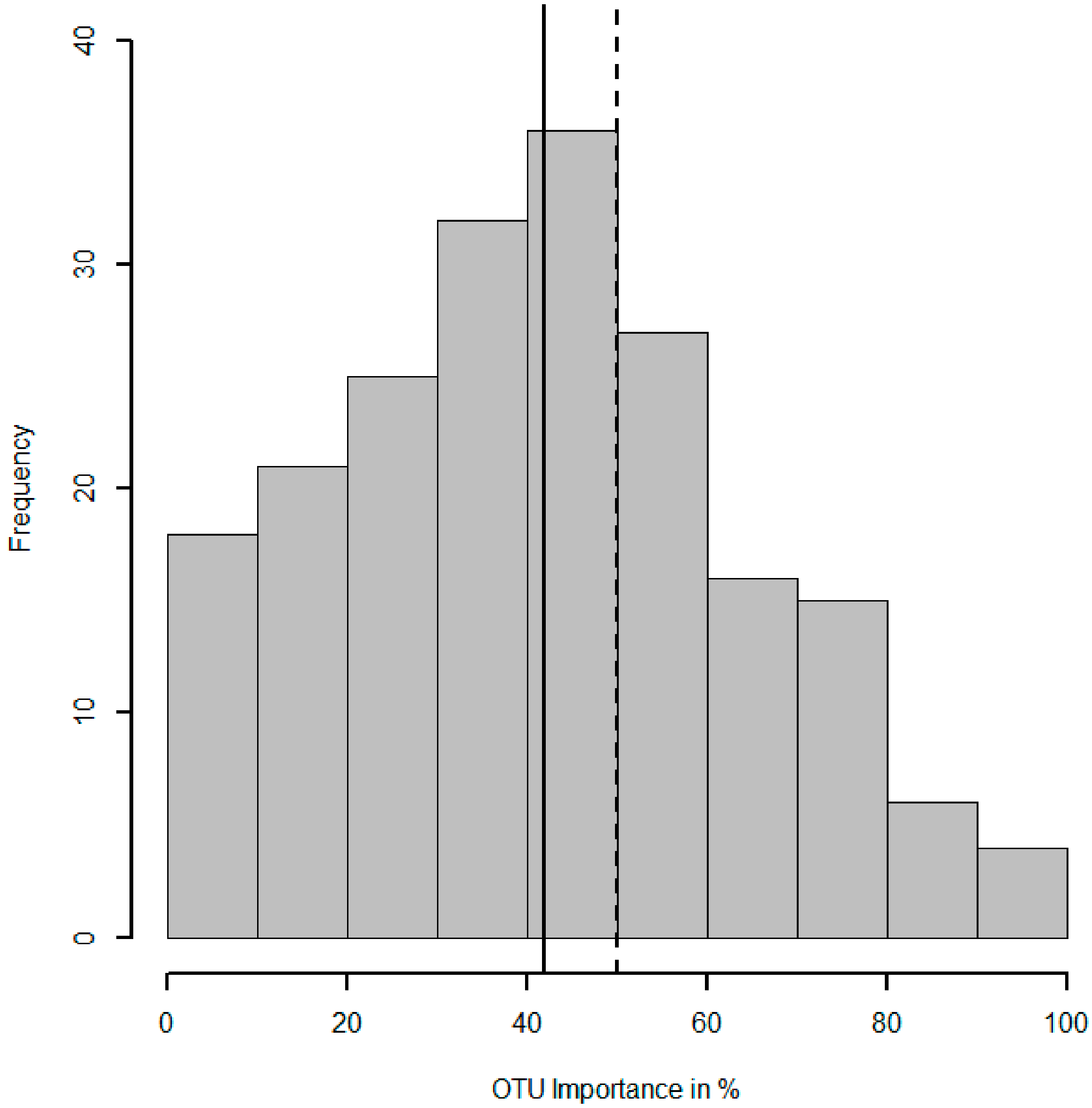

Figure S6. Importances of the 200 OTUs used in the neural network model training. Mean (50 %) and median (41.9 %) importances are indicated with dashed and solid vertical lines, respectively, suggesting that the distribution is biased towards lesser importances.
